# Alzheimer's Amyloid-*β* Accelerates Cell Senescence and Suppresses the SIRT1/NRF2 Pathway in Human Microglial Cells

**DOI:** 10.1155/2022/3086010

**Published:** 2022-08-17

**Authors:** Yuqian An, Yi Li, Yujun Hou, Shichao Huang, Gang Pei

**Affiliations:** ^1^State Key Laboratory of Cell Biology, CAS Center for Excellence in Molecular Cell Science, Shanghai Institute of Biochemistry and Cell Biology, Chinese Academy of Sciences, University of Chinese Academy of Sciences, Shanghai, China; ^2^School of Life Science and Technology, ShanghaiTech University, Shanghai, China; ^3^Institute for Regenerative Medicine, Shanghai East Hospital, Shanghai Key Laboratory of Signaling and Disease Research, School of Life Sciences and Technology, Tongji University, Shanghai, China; ^4^Shanghai Key Laboratory of Signaling and Disease Research, Collaborative Innovation Center for Brain Science, School of Life Sciences and Technology, Tongji University, Shanghai, China; ^5^Institute for Stem Cell and Regeneration, Chinese Academy of Sciences, Beijing, China

## Abstract

Microglia play important roles in maintenance of brain homeostasis, while due to some pathological stimuli in aging-related neurodegenerative diseases including Alzheimer's disease, they are malfunctioning. Here, we demonstrated that amyloid-*β* (A*β*) accelerated cell senescence characterized by the upregulation of p21 and PAI-1 as well as senescence-associated beta-galactosidase (SA-*β*-gal) in human microglial cells. Consistently, A*β* induced the senescence-associated mitochondrial dysfunctions such as repression of ATP production, oxygen consumption rate (OCR), and mitochondrial membrane potential and enhancement of ROS production. Furthermore, A*β* was found to significantly suppress mRNA expression and protein level of Sirtuin-1 (SIRT1), a key regulator of senescence, and inhibit mRNA expression and translocation of NRF2, a critical transcription factor in inflammatory responses, leading to impairment of phagocytosis. Rescue of SIRT1, as expected, could counteract the pathological effects of A*β*. In summary, our findings revealed that A*β* accelerates human microglial senescence mainly through its suppression of the SIRT1/NRF2 pathway and suggested that genetic and pharmaceutical rescue of SIRT1 may provide a potential alternative treatment.

## 1. Introduction

Alzheimer's disease (AD) is an age-related neurodegenerative disease and is often characterized by tau aggregation and amyloid-*β* (A*β*) deposition [1, 2]. It is identified that accumulation and aggregation of A*β* drives subsequent pathological events such as neuroinflammation, mitochondrial dysfunction, and cell senescence [3–5]. Microglia are the brain's innate immune cells and play important roles in AD [6, 7]. Compelling evidence suggests that microglia, in the aged neurodegenerative brain, are activated and recruited to A*β* plaques and secreted proinflammatory cytokines such as IL-1*β*, IL-6, and TNF-*α*, which are similar to immunosenescence of macrophages [8]. Cell senescence affects phagocytosis of microglial in AD mice [9]. Cell senescence also leads to microglia dysfunction, resulting in inaccurate response to external stimuli and neurodegeneration worsening [8, 10, 11]. The prominent feature of microglial senescence includes the morphological alteration described as “dystrophy,” [12] telomere shortening [13, 14], and functional alterations [8]. During senescence, microglia shift the glycolytic metabolic state featured by the mitochondrial activity [3, 15], change their inflammatory profile, increase the immunophenotypic expression, and more importantly, switch from neuroprotective to neurotoxic role when activated [10, 16–18]. A*β*, the main contributor of AD, has been suggested to accelerated microglial senescence [13]. However, there is still no direct evidence so far showing the influence of A*β* deposition on human microglial senescence.

Sirtuin-1 (SIRT1) is a NAD-dependent deacetylase that participates in the regulation of cell senescence, metabolism, inflammation, and mitochondrial function [19, 20]. Under homeostasis, the expression and activity of SIRT1 is controlled by multiple mechanisms and maintained at normal state [21, 22]. However, during aging, metabolic disorder, or neurodegenerative diseases, the expression of SIRT1 is diminished, intensifying oxidative stress potentially [23–25]. It is well known that sharp decrease of SIRT1 level is closely related to the accumulation of A*β* and tau proteins in AD patients [26–28]. SIRT1 has been suggested to reduce A*β* deposition and toxicity and improved AD pathology based on several earlier *in vitro* and *in vivo* studies [29–31]. Furthermore, SIRT1 is closely associated with nuclear factor E2-related factor 2 (NRF2), a transcription factor involved in regulating inflammatory responses through activating its downstream genes [32–34]. Therefore, regulation of the SIRT1/NRF2 pathway may provide a hopeful way for preventing or treating aging-related neurodegenerative disease.

Here, we used A*β* to induce cellular senescence in human microglial cells. After A*β* stimulation, we found the senescence-related mitochondrial functions were exacerbated significantly. We also detected that A*β* induction affected phagocytosis and ROS production of microglia and downregulated the SIRT1/NRF2 pathway. Overexpression of SIRT1 or using SIRT1 activator such as aspirin can counteract A*β*-induced cellular senescence. In summary, our results suggest that the SIRT1/NRF2 pathway is a therapeutic target for AD-related cellular senescence.

## 2. Materials and Methods

### 2.1. Cell Culture

Human microglial cells HMC3 were obtained from ATCC (#CRL0314). Cells were cultured in MEM with 10% FBS and 1% penicillin-streptomycin in a humidified incubator with 5% CO_2_/95% air at 37°C.

### 2.2. A*β* Peptide Preparation

The A*β* peptides (Chinese peptide) were prepared according to the protocols described previously [34, 35]. In brief, A*β* peptides were dissolved in HFIP (Sigma, #105228) to a final concentration of 1 mM, the HFIP-treated A*β* peptides were resolved in DMSO and then diluted to a concentration of 100 *μ*M with DMEM/F12 phenol-red free medium and incubated at 4°C for 24 h. After centrifugation at 12,000 g for 10 min, the supernatant with soluble A*β* was added to cultures. A*β*_42-1_ peptides (Beyotime, #P9005) were used as a negative control. A*β*_42-1_ peptides were prepared using the same protocol. The concentration of A*β*_42-1_ was 10 *μ*M. In this paper, A*β* presented A*β*_1-42_.

### 2.3. SA-*β*-Gal Staining

Senescence-associated *β*-galactosidase (SA-*β*-gal) activity was performed using the SA-*β*-gal staining kit (Beyotime, #C0602), according to the manufacturer's instructions. In brief, cells were plated in the density of 40,000 cells per well into a 24-well plate. After 24 h seeding, cells were treated with A*β* for 72 h, then the cells were fixed with 4% formaldehyde in PBS for 15 min, and the fixed cells were stained with SA-*β*-Gal staining solution at 37°C for 15 h. The percentage of positively stained cells were calculated based on three replicates.

### 2.4. Reverse Transcription and Quantitative Real-Time PCR

HMC3 cells were stimulated with A*β* for 72 h; then, the cells were extracted by TRI Reagent (Sigma, #T9424) to obtain total RNA according to the manufacturer's instructions. cDNA was synthesized using cDNA Synthesis kit (TaKaRa, #RR036B) and qPCR analysis was done with power SYBR Green PCR master mix (Vazyme, #Q712). Primers used were as follows: *PAI-1* (forward: 5′-ACCGCAACGTGGTTTTCTCA-3′ and reverse: 5′-TTGAATCCCATAGCTGCTTGAAT-3′), *p21* (forward: 5′-CGAAGTCAGTTCCTTGTGGAG-3′ and reverse: 5′-AGTCGTGGTCTTTG GGAGTC-3′), *CCNA1* (forward: 5′-GAAATTGTGCCTTGCCTGAGTG-3′ and reverse: 5′-TCTGATATGGAGGTGAAGTTCTGGA-3′), *CCND1* (forward: 5′-ATGTTCGTGGCCTCTA AGATGA-3′ and reverse: 5′-CAGGTTCCACTTGAGCTTGTTC-3′), *SIRT1* (forward: 5′-TAG CCTTGTCAGATAAGGAAGGA-3′ and reverse: 5′-ACAGCTTCACAGTCAACTTTGT-3′), *SIRT5* (forward: 5′-GCCATAGCCGAGTGTGAGAC-3′ and reverse: 5′-CAACTCCACAAGA GGTACATCG -3′), *NRF2* (forward: 5′-TCAGCGACGGAAAGAG TATGA-3′ and reverse: 5′- CCACTGGTTTCTGACTGGATGT-3′), *TNFα* (forward: 5′-CCTCTCTCTAATCAGCCCTCT G-3′ and reverse: 5′-GAGGACCTGGGAGTAGATGAG-3′), *IL1β* (forward: 5′-ATGATGGCT TATTACAGTGGCAA-3′ and reverse: 5′-GTCGGAGATTCGTAGCTGGA-3′), *IL6* (forward: 5′-ACTCACCTCTTCAGAACGAATTG-3′ and reverse: 5′-CCATCTTTGGAAGGTTCAGGT TG-3′), and *HPRT* (forward: 5′-CCTGGCGTCGTGATTAGTGAT-3′ and reverse: 5′-AGACGTTC AGTCCTGTCCATAA-3′). The reaction parameters were as follows: 95°C for 10 min; 95°C for 30 s, 40 cycle; 60°C for 30 s; and 72°C for 30 s. An additional cycle was performed for evaluation of primer's dissociation curve: 95°C for 1 min, 60°C for 30 s, and 95°C for 30 s. 2^–*ΔΔ*CT^ was used to analyze expression of genes. The gene levels were to HPRT endogenous control.

### 2.5. Western Blotting

Western blotting was performed as described previously [34]. Briefly, 20 *μ*g samples were loaded and separated on 10% or 12% SDS-PAGE and transferred to PVDF membranes. Membranes were blocked with 5% milk in TBST for 1 h, we used TBST to dilute primary and secondary antibodies. Membranes were incubated with primary antibody overnight at 4°C, washed in TBST, and incubated with HRP-conjugated secondary antibody for 60 min. The proteins of interest were performed using an ECL western blot detection kit (Bio-Rad). ImageJ software was used to evaluate the densitometry. Actin or proliferating cell nuclear antigen (PCNA) was used as loading control. Antibodies used were as follows: p53 (Beyotime, #AF7671), PAI-1 (Cell Signaling, #49536), p21 (Cell Signaling, #2947), SIRT1 (Cell Signaling, #8469), SIRT5 (Cell signaling, #8779), NRF2 (ABclonal, #A0674), PCNA (Cell Signaling, #13110), and Actin (Cell Signaling, #3700).

### 2.6. Measurement of Membrane Mitochondrial Potential (MMP)

HMC3 cells were plated at 9,000 cells per well in black-walled 96-wells plates and cultured overnight, then stimulated with 10 *μ*M A*β* for 72 h. JC-1 kit (Beyotime, #C2006) was used to detect the MMP level of cells according to the manufacturer's instructions. In brief, cells were loaded with JC-1 staining solution for 30 min at 37°C and then washed with staining buffer for twice. The cells were captured using Zeiss confocal laser scanning microscope (Zeiss 880 Airyscan). The fluorescence intensity was measured at 490/530 nm (green) for monomers and 525/590 nm for aggregates (red) using the BioTek SynergyNEO (BioTek), and the ratio of red/green fluorescence intensity was presented as MMP.

### 2.7. ROS Production

DCFH-DA (Beyotime, #S0033) was used to assess intracellular ROS levels. Briefly, HMC3 cells were seeded into black-walled 96-well plate at 9,000 cells/well density. Cells were stimulated with 10 *μ*M A*β* for 72 h and followed by staining with 10 *μ*M DCFH-DA in PBS for 30 min at 37°C. PBS was used to wash the cells for three times. Then, the cells were detected using Zeiss confocal laser scanning microscope (Zeiss 880 Airyscan). Lastly, fluorescence was measured at 485 nm excitation/538 nm emission using a BioTek SynergyNEO (BioTek, USA), and the fluorescence signal was normalized to the Hoechst.

### 2.8. Measurement of Oxygen Consumption Rate (OCR)

Oxygen consumption rate (OCR) was measured using a Seahorse XF24 analyzer (Seahorse Bioscience) according to the manufacturer's guidance. Briefly, 9,000 cells were plated on the XF24 cell culture microplate and cultured with 10 *μ*M A*β* for 72 h. Then, the cells were washed twice and maintained in XF assay medium. OCR was measured under basal condition and also after the injection of oligomycin (1 *μ*M), FCCP (1 *μ*M), rotenone (1 *μ*M), and antimycin A (1 *μ*M). After baseline measurements, oligomycin (1 *μ*M), FCCP (1 *μ*M), rotenone (1 *μ*M), and antimycin A (1 *μ*M) was injected sequentially. Data were analyzed using Seahorse XF24 Wave software, and the results were normalized to cell number.

### 2.9. Phagocytosis Assay

HMC3 cells were plated into 96-well plates at 9,000 cells per well and cultured overnight and then treated with A*β* for 72 h. After that, the cells were washed with PBS and the medium were changed to FBS-free DMEM alone at 37°C for 6 h. The fluorescent latex beads (Sigma, #L1030) were preopsonised in 50% FBS and PBS, and the beads were loaded to the cells at concentrations of 20 beads per cell and incubated at 37°C for 3 h. After A*β* uptake, the cells were processed for immunofluorescence using Zeiss confocal laser scanning microscope (Zeiss 880 Airyscan). Hoechst was used to stain the nuclei. Lastly, the fluorescence intensity was also detected using the BioTek SynergyNEO (BioTek, USA) at 485 nm excitation/538 nm emission.

### 2.10. Nuclear and Cytoplasmic Extraction

HMC3 cells were cultured in 6 cm plates, grew for 24 h, and then were treated with A*β* for indicated time. Nuclear and Cytoplasmic Protein Extraction Kit (Beyotime, #P0028) was used in this experiment according to the manufacturer's instructions. In brief, 80 *μ*l Buffer A was added to cells for 10 min, next 5 *μ*l Buffer B was added, then the cells were centrifugated at 12,000 g for 5 min, and the supernatant was the cytoplasmic protein. The precipitation was resolved with 25 *μ*l Buffer C for 30 min and centrifuged at 16,000 g for 10 min at 4°C to obtain the nuclear fraction.

### 2.11. SIRT1 Overexpression

SIRT1 cDNA was made from pCMV-SIRT1-t1-Flag (purchased from Sino Biological) via PCR amplification. SIRT1 cDNA was cloned into the FUGW vector using Seamless Cloning Kit (Beyotime, D7010M) and confirmed by DNA sequencing. HMC3 cells were plated into 24-well or 6 cm dish at appropriate intensity and cultured overnight. We transfected 150 ng plasmid per well into 24-well or 1.5 *μ*g plasmid per well into 6 cm dish. SIRT1 plasmid or FUGW plasmid was transfected using ViaFect reagent (Promega, #E4981) according to manufacturer's instructions. After transfection for 24 h, cells were stimulated by A*β* for 72 h. The knockdown of SIRT1 was performed by the transfection with specific siRNA (Tsingke Biotechnology Co., Ltd.) using ViaFect reagent. The cloning primers used were as follows: SIRT1 (forward: 5′-TGGGCTGCAGGTCGACTCTAGAATGGCAGATGAAGCAGCTCTC-3′ and reverse: 5′-TTG ATATCGAATTCTAGACTATGATTTGTTTGATGGATAGTTCATGTCT-3′). The siRNA primers were as follows: siSIRT1-1 (forward: 5′-CACCUGAGUUGGA UGAUAUTT-3′ and reverse: 5′-AUAUCAUCCAACUCAGGUGTT-3′) and siSIRT1-2 (forward: 5′-GUCUGUUUCAUG UGGAAUATT-3′and reverse: 5′-UAUUCCACAUGAAACAGACTT-3′).

### 2.12. Statistical Analysis

GraphPad Prism 7.0 was used to draw graphs and perform data analysis. The data are presented as mean ± SEM, *n* ≥ 3 independent experiments; ^∗^*p* < 0.05, ^∗∗^*p* < 0.01, ^∗∗∗^*p* < 0.001, and ^∗∗∗∗^*p* < 0.0001, analyzed by one-way ANOVA followed by Bonferroni's test.

## 3. Results

### 3.1. A*β* Induced Senescence Gene Activation in Human Microglial Cells

**Figure 1:**
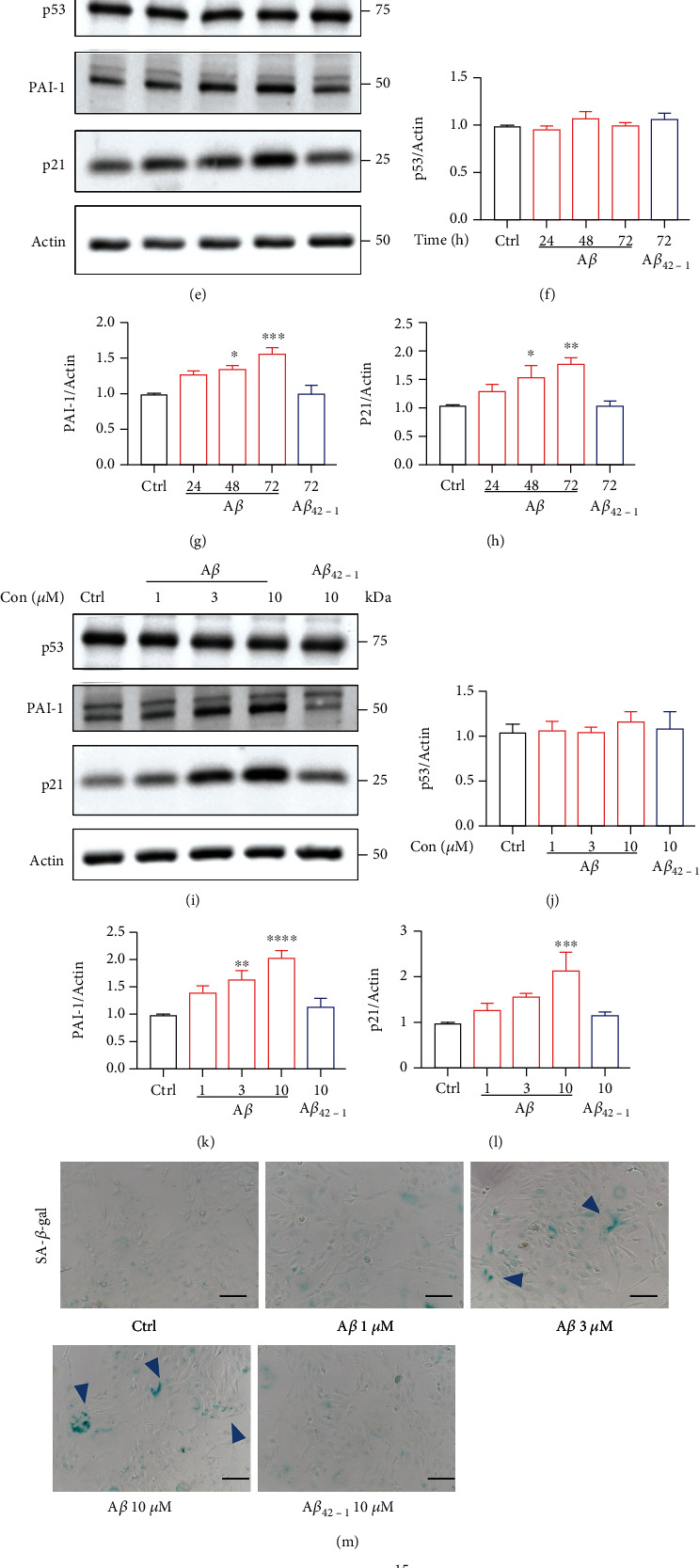
A*β*-induced gene expression of senescence in human microglial cells. (a–h) Human microglial cells were treated with 10 *μ*M A*β* for different times. (a–d) Relative genes expression levels of *PAI-1*, *p21*, *CCND1*, and *CCNA1* were measured by qPCR. (e) Protein levels of p53, PAI-1, and p21 were detected by western blotting. (f–h) The quantification of relative protein levels in (e). (i–n) Human microglial cells were stimulated with A*β* (1-10 *μ*M) for 72 h. (i) Western blotting was used to investigate protein levels of p53, PAI-1, and p21. (j–l) The quantification of relative protein levels in (i). (m) The representative SA-*β*-gal staining images. (n) Quantification of the percentage of SA-*β*-gal cells. Scar bar, 100 *μ*m. (o–q) Senescence-associated proinflammatory cytokines such as *TNF-α*, *IL-1β*, and *IL-6* were detected by qPCR. The data are presented as mean ± SEM, *n* ≥ 3 independent experiments; ^∗^*p* < 0.05, ^∗∗^*p* < 0.01, ^∗∗∗^*p* < 0.001, and ^∗∗∗∗^*p* < 0.0001, analyzed by one-way ANOVA followed by Bonferroni's test.

Several studies have shown strong evidences that cellular senescence increased significantly in AD mice [33, 35, 36]. Here, we treated human microglial cells HMC3 with 10 *μ*M A*β* for different times and evaluated the gene expression of senescence. Compared with the A*β*_42-1_ treatment control group, cells treated with A*β* displayed significantly higher expression of senescence-associated genes (Figures 1(a)–1(d)). Similarly, the protein level of p53, PAI-1, and p21 was detected, showing that PAI-1 and p21 was markedly increased at 72 h with 10 *μ*M A*β* stimulation, but there was no obvious change about the level of p53 (Figures 1(e)–1(h)). Thus, treatment of A*β* for 72 h was applied for the subsequent experiments. We also measured the effects of different A*β* concentrations and found that A*β* increased the protein level of PAI-1 and p21 significantly at 10 *μ*M (Figures 1(i)–1(l)). Furthermore, we performed a senescence-associated beta-galactosidase (SA-*β*-gal) assay to confirm the senescence phenotype of HMC3 cells. As shown in Figures 1(m) and 1(n), an increased percentage of SA-*β*-gal-positive cells is observed in cell culture treated with 10 *μ*M A*β*. We also evaluated SASP markers such as *TNF-α*, *IL-1β*, and *IL-6* by qPCR (Figures 1(o)–1(q)).These results indicated that A*β* induced senescence in human microglial cells.

### 3.2. A*β* Accelerated Mitochondrial Dysfunction in Microglia

**Figure 2:**
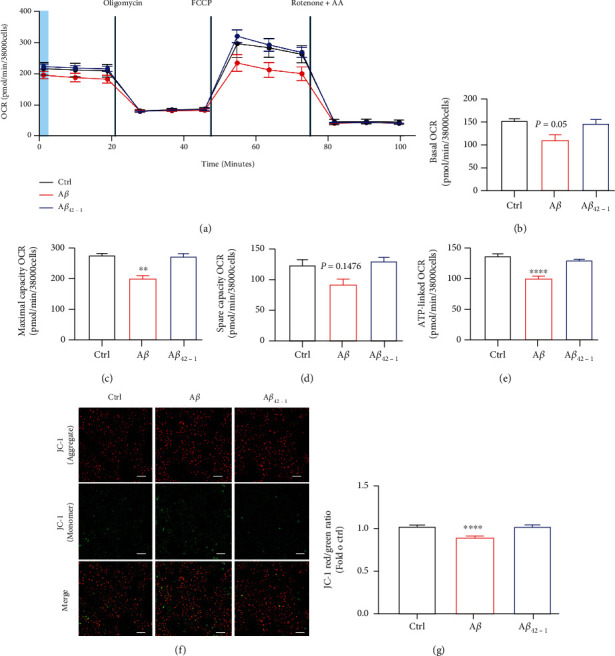
A*β* accelerated microglial mitochondrial dysfunctions. (a–e) A*β*-induced reduction of oxygen consumption rate (OCR) in HMC3 cells. Seahorse assays showed mitochondrial bioenergetics in HMC3 cells with 10 *μ*M A*β* for 72 h. (a) The representative graph of the mitochondrial stress test detailing the four key parameters of mitochondrial function through sequential addition of oligomycin (1 *μ*M), FCCP (1 *μ*M), and rotenone/antimycin A (1 *μ*M each), which allowed the measurement of basal respiration (b), the maximal respiration (c), the spare respiratory capacity (d), and mitochondrial ATP production (e). (f, g) HMC3 cells were treated with 10 *μ*M A*β* for 72 h. The cells were stained with JC-1 dye and then captured by Zeiss 880 microscope (f), and the fluorescence intensity in (f) was quantified using BioTek reader (g). Scale bars, 100 *μ*m. The data are presented as mean ± SEM, *n* ≥ 3 independent experiments, ^∗∗^*p* < 0.01 and ^∗∗∗∗^*p* < 0.0001, analyzed by one-way ANOVA followed by Bonferroni's test.

Recent studies revealed that cellular senescence is associated with mitochondrial defects [37–39]. We therefore assessed the effects of A*β* on mitochondrial functions in HMC3 cells. To evaluate mitochondrial functions, we tested the oxygen consumption rate (OCR) in HMC3 after treatment with oligomycin (ATP synthase inhibitor), FCCP (H+ ionophore), or rotenone and antimycin A (electron-transport chain inhibitor). These results revealed that A*β* treatment significantly diminished the maximal respiratory capacity of mitochondria and ATP production in microglia compared with vehicle. A*β* treatment also reduced basal respiration and spare capacity OCR, but no statistically significant change was observed (Figures 2(a)–2(e)), A*β*_42-1_, as the negative control, had no obvious effect. Furthermore, we tested whether A*β* could induce the loss of mitochondrial membrane potential (MMP). In this study, we used JC-1 probe to evaluate MMP in HMC3 cells. Red fluorescence and green fluorescence characterized high and low mitochondrial membrane permeability, respectively, and the ratio could signify the change of MMP. Cells treated with A*β* increased green fluorescence intensity (Figure 2(f)) and reduced the red/green fluorescence (Figure 2(g)), indicating A*β* induced depolarization. Taken together, our data indicated that A*β* induced mitochondrial dysfunctions through reduction of OCR and MMP.

### 3.3. A*β* Decreased Microglial Phagocytosis and Increased ROS Production

**Figure 3:**
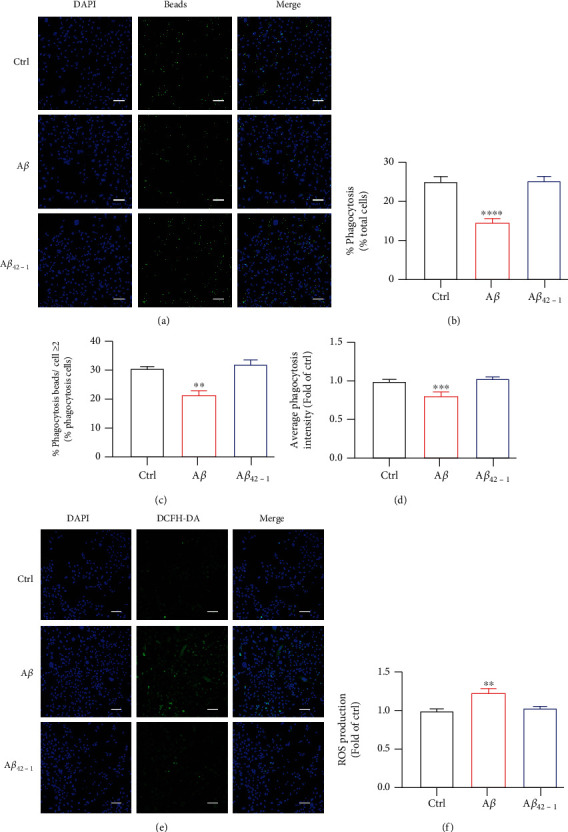
A*β* decreased microglial phagocytosis and increased ROS production. (a–d) HMC3 cells were mixed with the fluorescent latex beads. The cells were captured by Zeiss 880 microscope (a). Scale bars, 100 *μ*m. (b, c) The quantification of in (a). The fluorescence intensity was also quantified using BioTek reader (d). (e, f) ROS production in HMC3 cells was investigated with the DCFH-DA probe. The pictures were obtained by Zeiss microscope. Scale bars, 100 *μ*m. The fluorescence intensity in (e) was quantified using BioTek reader (f). The data are presented as mean ± SEM, *n* ≥ 3 independent experiments; ^∗∗^*p* < 0.01, ^∗∗∗^*p* < 0.001, and ^∗∗∗∗^*p* < 0.0001, analyzed by one-way ANOVA followed by Bonferroni's test.

Phagocytosis, one of the most important feature of microglia, has been reported to be decreased significantly in AD mice [3, 40–42]. Moreover, recent studies showed that the phagocytic activity of mouse primary microglial cells was markedly decreased with A*β* stimulation, which is associated with mitochondrial dysfunction concluding reduction of OCR [3, 42]. However, whether A*β* could affect phagocytosis in human microglial cells is unknown. Here, we treated HMC3 cells with A*β* for 72 h and mixed with fluorescent latex beads for 3 h. The phagocytic capacity was assessed by confocal microscope (Figures 3(a)–3(c)). And also, the average cell fluorescence intensity was detected at 485 nm excitation/538 nm emission using the a BioTek fluorescence reader (Figure 3(d)). A*β* also decreased human microglial paghocytosis by flow cytometry (Supplementary Figure 1A). The results revealed A*β* significantly reduced phagocytic capacity (Figures 3(a)–3(d)). Inflammatory responses, another vital feature of microglia, increase dramatically in AD mice and AD patients. A*β* could induce ROS generation, thus causing oxidative stress of microglia. Here, we stimulated human microglial cells with A*β* for 72 h and assessed the intracellular ROS level by staining with the DCFH-DA probe. The probe has no fluorescence and can pass through plasma membrane freely and produce fluorescent DCF when oxidized by ROS. Results showed that treatment with A*β* markedly increased DCF fluorescence (Figures 3(e) and 3(f)). Taken together, these results indicated A*β* significantly impaired phagocytic capacity and increased ROS production in human microglia cells.

### 3.4. A*β* Downregulated the SIRT1/NRF2 Pathway in the Cells

**Figure 4:**
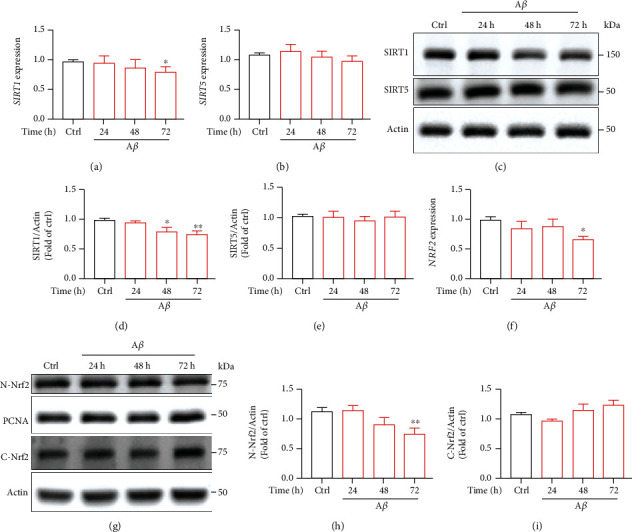
A*β* downregulated the SIRT1/NRF2 pathway in the cells. (a–i) HMC3 microglia cells were treated with 10 *μ*M A*β* for different times. (a, b) mRNA expression of *SIRT1* and *SIRT5*. (c) Protein level of SIRT1 and SIRT5 was detected by western blotting. (d, e) The quantification of relative protein levels in (c). (f) mRNA expression of *Nrf2.* (g) Protein levels of N-Nrf2 and C-Nrf2 were observed by western blotting. (h, i) The quantification of relative protein levels in (g). The data are presented as mean ± SEM, *n* ≥ 3 independent experiments; ^∗^*p* < 0.05 and ^∗∗^*p* < 0.01, analyzed by one-way ANOVA followed by Bonferroni's test.

SIRT1, which is a NAD+-dependent deacetylase, has been reported to play an important role in age-related neurodegenerative diseases [19, 43, 44]. Recent studies have shown that the expression of SIRT1 was decreased markedly in AD patients [26–28]. Here, we wanted to understand whether SIRT1 took part in A*β*-induced microglial senescence. We treated cells with A*β* for different times and found that at 72 h, A*β* reduced the mRNA expression of *SIRT1* and downregulated the protein level of SIRT1 (Figures 4(a)–4(e)). SIRT5, another SIRT family protein, was not affected after A*β* stimulation. Furthermore, accumulating evidence showed that SIRT1 is involved in the activation of nuclear factor E2-related factor 2 (NRF2) [32, 33]. NRF2 can be served as a sensor of oxidative stress. Next, we investigated whether A*β* treatment affected nuclear translocation of NRF2. The results indicated that A*β* reduced the mRNA expression of *NRF2* (Figure 4(f)). Moreover, A*β* inhibited NRF2 nuclear translocation (N-Nrf2) in a time-dependent manner (Figures 4(g) and 4(h)), NRF2 levels in the cytoplasm (C-Nrf2) were not statistically changed (Figures 4(g) and 4(i)). In conclusion, the downregulated SIRT1/NRF2 pathway accelerated cellular senescence in human microglia. Transfected of HMC3 cells with siSIRT1-1 or siSIRT1-2 downregulated protein level of SIRT1 detected by western blot (Supplementary Figure 2A and B). The knockdown of SIRT1 resulted in a significant increase in the percentage of SA-*β*-gal-positive cells in the cells (Supplementary Figure 2C and D). These results detected that SIRT1 may be involved in human microglial senescence.

### 3.5. Overexpression of SIRT1 Rescues A*β*-Induced Senescence and Mitochondrial Dysfuntions

**Figure 5:**
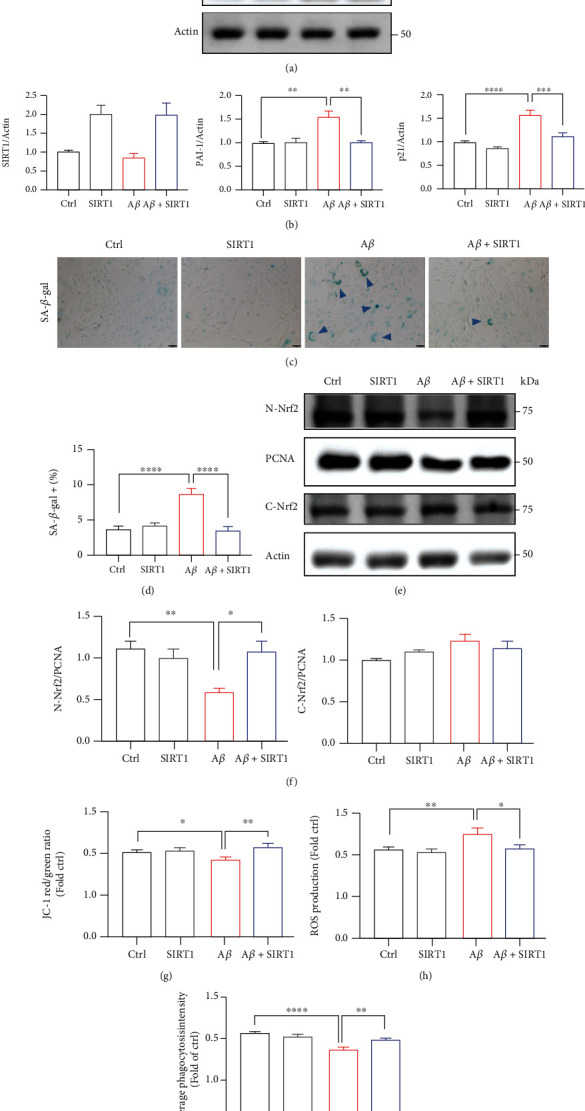
Overexpression of SIRT1 rescues A*β*-induced senescence and mitochondrial dysfunctions. (a–i) Human microglia cells HMC3 were transfected with SIRT1 plasmid (150 ng) or FUGW (150 ng) followed by A*β* treatment for 72 h. (a) Protein levels of SIRT1, PAI-1, and p21 were investigated by western blotting. (b) The quantification of relative protein levels in (a). (c) The representative SA-*β*-gal staining pictures. (d) Quantification of the percentage of SA-*β*-gal cells. Scar bar, 50 *μ*m. (e) Nuclear Nrf2 and Cyto Nrf2 were detected after SIRT1 overexpression. (f) The quantification of relative protein levels in (e). (g) The cells were incubated with JC-1 dye and the fluorescence intensity was quantified using BioTek reader. (h) HMC3 cells were stained with DCFH-DA and Hoechst; then, the fluorescence intensity was detected by BioTek reader. (i) The fluorescent latex beads were added to the medium and incubated at 37°C for 3 h. The fluorescence intensity was quantified using BioTek reader. The data are presented as mean ± SEM, *n* ≥ 3 independent experiments; ^∗^*p* < 0.05, ^∗∗^*p* < 0.01, ^∗∗∗^*p* < 0.001, and ^∗∗∗∗^*p* < 0.0001, analyzed by one-way ANOVA followed by Bonferroni's test.

Since the protein level of SIRT1 has been reduced with A*β* treatment, we next tested whether overexpression of SIRT1 could rescue A*β* defects including senescence, mitochondrial disability, and microglial dysfunctions. Here, we transfected HMC3 cells with SIRT1 plasmid or FUGW plasmid. As shown in Figures 5(a) and 5(b), A*β* markedly downregulated SIRT1 expression and upregulated senescence genes including PAI-1 and p21, but SIRT1 overexpression almost counteracted the influence of A*β*-induced senescence in HMC3 cells (Figures 5(a) and 5(b)). Furthermore, SIRT1 overexpression decreased the SA-*β*-gal signal in the cells relative to the vehicle controls (Figures 5(c) and 5(d)). We next examined whether overexpression of SIRT1 could promote NRF2 nuclear translocation. Cells transfected with SIRT1 translocated NRF2 to the nucleus (Figures 5(e) and 5(f)). Interestingly, overexpression of SIRT1 prevented A*β* impaired mitochondrial membrane potential (Figure 5(g) and Supplementary Figure 3A). Similarly, A*β*-induced ROS production was significantly rescued by SIRT1 overexpression (Figure 5(h) and Supplementary Figure 3B). Moreover, SIRT1 treatment involved in the enhancement of A*β* phagocytosis (Figure 5(i) and and Supplementary Figure 3C). Together, these data suggested that SIRT1 protein was dispensable for the A*β*-mediated cell senescence, mitochondrial dysfunctions, and microglial state.

### 3.6. Aspirin Alleviates A*β*-Induced Senescence and Mitochondrial Dysfunctions via Upregulation of SIRT1 Pathway

**Figure 6:**
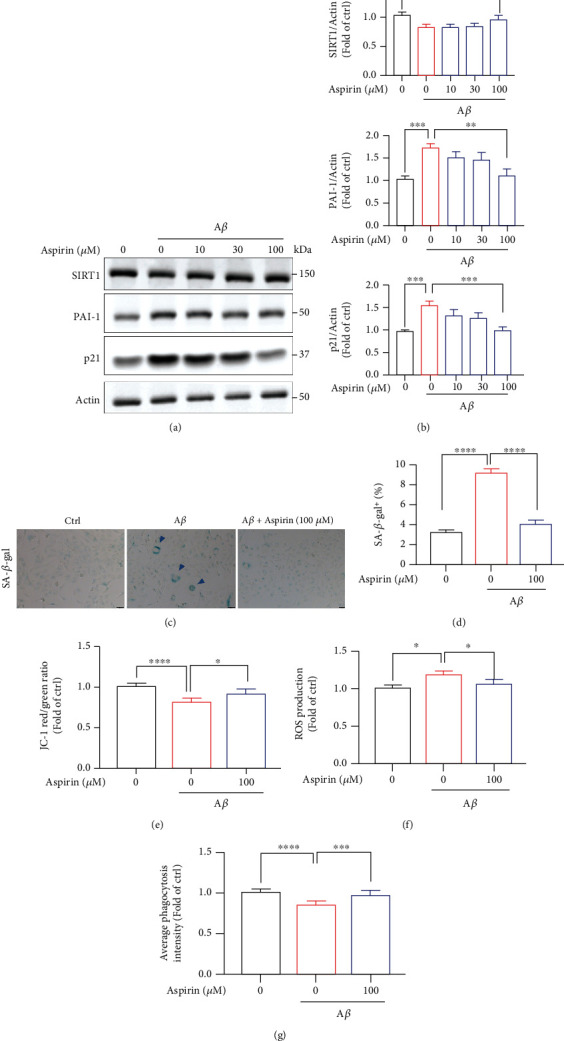
Aspirin alleviates A*β*-induced senescence and mitochondrial dysfunctions via upregulation of SIRT pathway. (a–g) HMC3 cells were preincubated with aspirin for 4 h followed by treatment with 10 *μ*M A*β* for 72 h. (a) Protein levels of SIRT1, PAI-1, and p21 were detected by western blotting. (b) The quantification of relative protein levels in (a). (c) The representative SA-*β*-gal staining images. (d) Quantification of the percentage of SA-*β*-gal cells. Scar bar, 50 *μ*m. (e) The cells were stained with JC-1 dye and the fluorescence intensity was quantified using BioTek reader. (f) HMC3 cells were incubated with DCFH-DA and Hoechst; then, the fluorescence intensity was detected by BioTek reader. (g) The fluorescent latex beads were added to the medium and incubated at 37°C for 3 h. The fluorescence intensity was quantified using BioTek reader. The data are presented as mean ± SEM, *n* ≥ 3 independent experiments; ^∗^*p* < 0.05, ^∗∗^*p* < 0.01, ^∗∗∗^*p* < 0.001, and ^∗∗∗∗^*p* < 0.0001, analyzed by one-way ANOVA followed by Bonferroni's test.

In this paper, we wanted to find some drugs which could relieve microglial cellular senescence. Surprisingly, treatment of aspirin in HMC3 was found to rescue cellular senescence after A*β* stimulation (Figures 6(a) and 6(b)). Aspirin is a common drug, which was widely used for treating pain, fever, inflammation, and cardiovascular diseases [45–47]. Previous studies showed that aspirin could activate SIRT1 in liver cells and in endothelial cells [48, 49]. Here, pretreatment with aspirin for 4 h did lower senescence-associated protein levels obviously (Figures 6(a) and 6(b)). Meanwhile, 100 *μ*M aspirin markedly increased SIRT1 level. To assess the effect of aspirin on senescent microglia cells, SA-*β*-gal activity was detected and there was a significant reduction in the number of SA-*β*-gal-positive cells in aspirin-treated cells (Figures 6(c) and 6(d)). We also investigated whether aspirin could affect mitochondrial functions and found that aspirin increased mitochondrial membrane potential using JC-1 probe (Figure 6(e) and Supplementary Figure 4A). Lastly, 100 *μ*M aspirin significantly inhibited ROS production (Figure 6(f) and Supplementary Figure 4B) and increased phagocytic capacity in HMC3 cells (Figure 6(g) and Supplementary Figure 4C). In summary, aspirin may be a potential drug in aging-related neurodegenerative diseases through the SIRT1 pathway.

## 4. Discussion

AD is a neurodegenerative disease mainly characterized by the progressive aggregation of A*β* [50, 51]. The microglia play an important role in the maintenance of brain homeostasis [52, 53]. Recent studies indicated that microglia can be categorized into two opposite types: toxic phenotype and protective phenotype [54, 55]. Toxic microglia produce chemokines and cytokines such as CCL2, IL-1*β*, IL-6, IL-12, and TNF-*α* and generate nitrogen species and reactive oxygen. However, protective microglia produce anti-inflammatory cytokines such as IL-10 and TGF-*β* and growth factors. The dynamic changes of toxic/protective phenotypes are critically associated with AD. Endogenous stimuli including A*β* and tau may persistently activate proinflammatory responses and finally aggravate progression of neurodegenerative disease [35]. The expression of proinflammatory cytokines is one of the hallmarks of cellular senescence [37]. In the present study, we demonstrate that A*β* could induce microglial senescence. Srinivasan et al. revealed Alzheimer's patient microglia exhibited enhanced aging [56]. We also find that A*β* aggravate senescence-associated mitochondrial dysfunctions and impair microglial functions. Interestingly, we revealed that the SIRT1/NRF2 pathway is partly reduced by A*β* stimulation. Notably, overexpression of SIRT1 or use SIRT1 activator such as aspirin may rescue A*β* defects.

Increasing evidences point out that mitochondrial dysfunction is one of the hallmarks of aging [57, 58], attributing to the accumulation of mtDNA mutations, damaged fission and fusion behavior, weakened membrane potential, abnormal metabolism, and defective electron transport chain (ETC) function in mitochondrial. Mitochondrial dysfunctions in microglia has been linked to the development of aging-related neurodegenerative diseases such as AD [59–61]. The high level of reactive oxygen species (ROS) and loss of mitochondrial membrane potential have been observed in human microglial cells with A*β* stimulation in our work. Previous studies reported that a metabolic switch from mitochondrial OXPHOS to anaerobic glycolysis in A*β*-treated primary mouse microglia is associated with microglia phagocytosis [3, 42]. Here, we detected A*β* treatment significantly reduced OCR levels in human microglia cells and impaired the capacity of microglia phagocytosis. Thus, therapies targeting basic mitochondrial processes hold great promise.

Sirtuins are class III histone deacylases possessing outstanding properties in preventing diseases and reversing some aspects of aging [62–64]. SIRT1 has been shown to regulate cellular metabolism by acting as a cellular sensor [65]. Increasing studies indicate that the expression of SIRT1 is significantly diminished in aging, metabolic, and neurodegenerative diseases, leading to oxidative stress [66]. Importantly, the protein level of SIRT1 is also decreased dramatically in AD mice brains and AD patients, which is closely related to the accumulation of A*β* and tau proteins than in normal aging individuals [26, 67]. Here, we found SIRT1 was decreased after A*β* stimulation in human microglia cells. At the same time, SIRT1-associated NRF2 nuclear translocation was also reduced after A*β* treatment. Aspirin is one of the most widely used treatments for cardiovascular disease. Aspirin has obviously anti-inflammatory function. We found that aspirin could increase SIRT1 production and alleviate human microglial senescence. Aspirin has been reported to reduce amyloid plaque in a mouse model of AD [68]. However, aspirin does not reduce the risk of Alzheimer's disease in clinical trial [69]. There are many differences between cell lines and human. Therefore, activating the SIRT1/NRF2 pathway may provide a promising way for prevention and treatment of aging-related neurodegenerative diseases.

## Data Availability

The data, methods, and study materials used to conduct the research will be available from the corresponding authors on reasonable request.

## Supplementary Materials

Supplementary Materials Figure 1: A*β* decreased human microglial phagocytosis by flow cytometry. (A) Human microglia cells were mixed with the fluorescent latex beads. The cells were detected by flow cytometry. A*β*_42-1_ as a negative ctrl. Figure 2: knockdown SIRT1 accelerated cellular senescence in human microglial cells. (A, B) HMC3 cells were transfected with negative control (ctrl), siSIRT1-1, and siSIRT1-2 for 72 h. (A) SIRT1 protein level was detected by western blot. (B) Quantification of SIRT1 protein level in (A). (C) The representative images of SA-*β*-gal staining in HMC3 cells transfected with siSIRT1 stained after 72 h. (D) Quantification of SA-*β*-gal-positive cells in (C). Scale bars, 100 *μ*m. The data are presented as mean ± SEM, *n* ≥ 3 independent experiments, ^∗∗^*p* < 0.01 and ^∗∗∗∗^*p* < 0.0001, analyzed by one-way ANOVA followed by Bonferroni's test. Figure 3. Overexpression of SIRT1 rescues A*β*-induced mitochondrial dysfunction, ROS production, and phagocytic capacity. (A–C) HMC3 cells were transfected with SIRT1 plasmid (150 ng) or FuGW (150 ng) followed by A*β* treatment for 72 h. (A) JC-1 dye was used to detect mitochondrial membrane potential and then captured by Zeiss 880 microscope. Scale bars, 100 *μ*m. (B) HMC3 cells were stained with DCFH-DA probe to detect ROS production by Zeiss microscope. Scale bars, 100 *μ*m. (C) HMC3 cells were mixed with the fluorescent latex beads to detect phagocytic capacity. The cells were captured by Zeiss 880 microscope. Scale bars, 100 *μ*m. Figure 4. Aspirin alleviates A*β*-induced mitochondrial dysfunction, ROS production and phagocytic capacity. (A) HMC3 cells were stained with JC-1 dye and then captured by Zeiss 880 microscope. Scale bars, 100 *μ*m. (B) HMC3 cells were stained with DCFH-DA probe to detect ROS production by Zeiss microscope. Scale bars, 100 *μ*m. (C) HMC3 cells were mixed with the fluorescent latex beads. The cells were captured by Zeiss 880 microscope. Scale bars, 100 *μ*m.
