# Supplementary material for: Alzheimer's Amyloid-β Accelerates Cell Senescence and Suppresses the SIRT1/NRF2 Pathway in Human Microglial Cells
Source: Oxid Med Cell Longev. 2022 Aug 17;2022:3086010. doi: 10.1155/2022/3086010 (PMC9402294; doi:10.1155/2022/3086010)
Supplement: Supplementary Materials — Figure 1: Aβ decreased human microglial phagocytosis by flow cytometry. (A) Human microglia cells were mixed with the fluorescent latex beads. The cells were detected by flow cytometry. Aβ42-1 as a negative ctrl. Figure 2: knockdown SIRT1 accelerated cellular senescence in human microglial cells. (A, B) HMC3 cells were transfected with negative control (ctrl), siSIRT1-1, and siSIRT1-2 for 72 h. (A) SIRT1 protein level was detected by western blot. (B) Quantification of SIRT1 protein level in (A). (C) The representative images of SA-β-gal staining in HMC3 cells transfected with siSIRT1 stained after 72 h. (D) Quantification of SA-β-gal-positive cells in (C). Scale bars, 100 μm. The data are presented as mean ± SEM, n ≥ 3 independent experiments, ∗∗p < 0.01 and ∗∗∗∗p < 0.0001, analyzed by one-way ANOVA followed by Bonferroni's test. Figure 3. Overexpression of SIRT1 rescues Aβ-induced mitochondrial dysfunction, ROS production, and phagocytic capacity. (A–C) HMC3 cells were transfected with SIRT1 plasmid (150 ng) or FuGW (150 ng) followed by Aβ treatment for 72 h. (A) JC-1 dye was used to detect mitochondrial membrane potential and then captured by Zeiss 880 microscope. Scale bars, 100 μm. (B) HMC3 cells were stained with DCFH-DA probe to detect ROS production by Zeiss microscope. Scale bars, 100 μm. (C) HMC3 cells were mixed with the fluorescent latex beads to detect phagocytic capacity. The cells were captured by Zeiss 880 microscope. Scale bars, 100 μm. Figure 4. Aspirin alleviates Aβ-induced mitochondrial dysfunction, ROS production and phagocytic capacity. (A) HMC3 cells were stained with JC-1 dye and then captured by Zeiss 880 microscope. Scale bars, 100 μm. (B) HMC3 cells were stained with DCFH-DA probe to detect ROS production by Zeiss microscope. Scale bars, 100 μm. (C) HMC3 cells were mixed with the fluorescent latex beads. The cells were captured by Zeiss 880 microscope. Scale bars, 100 μm. [file 3086010.f1.zip › supplementary figure2 .pdf]

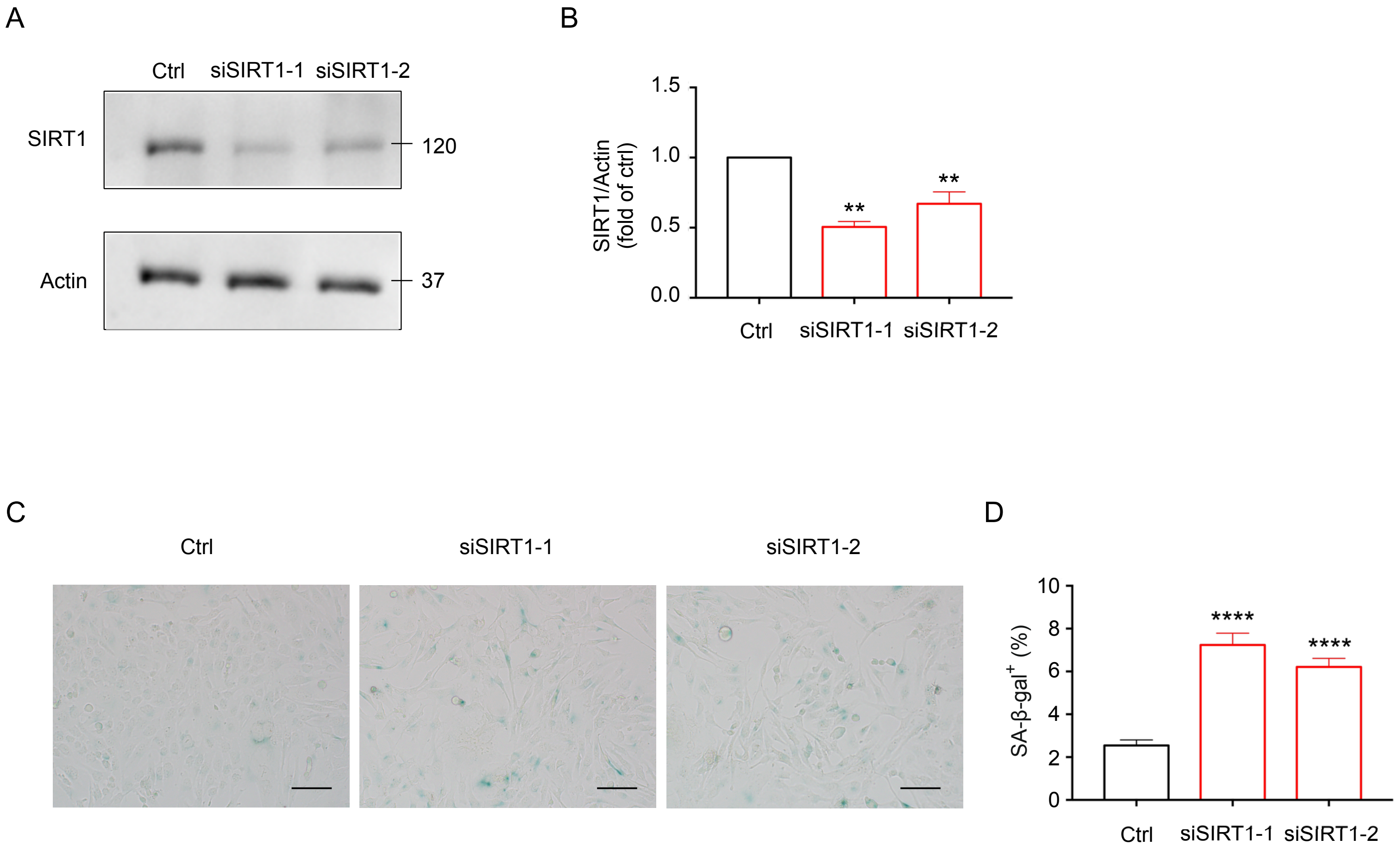

Supplementary Fig.2. Knock down SIRT1 accelerated cellular senescence in human microglial cells. (A and B) HMC3 cells were transfected with negative control (ctrl), siSIRT1-1, siSIRT1-2 for 72 h. (A) SIRT1 protein level was detected by western blot. (B) Quantification of SIRT1 protein level in A. (C) The representative images of SA-β-gal staining in HMC3 cells transfected with siSIRT1 stained after 72 h. (D) Quantification of SA-β-gal-positive cells in C. Scale bars, 100 μm. The data are presented as mean ± SEM, $n \geq 3$ independent experiments, $**p < 0.01$ and $****p < 0.0001$, analyzed by one-way ANOVA followed by Bonferroni's test.
